# Immunotherapy and tyrosine kinase inhibitors in chordoma: a real-world data study from a European Reference Network on Rare Adult Solid Cancers member center

**DOI:** 10.3389/fonc.2026.1755538

**Published:** 2026-02-03

**Authors:** Mario Balsa, Francesc Torrent, Diana Pérez, Alejandro Ruiz, Joan Maria Viñals, Oscar Pablos, Maria Fontalva, Federico Portabella, Alicia Lozano, Javier González-Viguera, Jose Antonio Narváez, Javier Hernández, Juan Carlos Sardiñas, Xavier Sanjuan, Gianni Ippoliti, Ma Rosa Comabella, Rosó Sala, Xavier García del Muro, Laura Jiménez, Juan Martin-Liberal

**Affiliations:** 1 Medical Oncology Department, Institut Català d'Oncologia (ICO), L’Hospitalet de Llobregat, Spain; 2 Clinical Research Unit, Institut Català d’Oncologia (ICO), L’Hospitalet De Llobregat, Spain; 3 Plastic Surgery Department, Hospital Universitari de Bellvitge (HUB), L’Hospitalet de Llobregat, Spain; 4 Orthopedic Surgery and Traumatology Department, Hospital Universitari de Bellvitge (HUB), L’Hospitalet de Llobregat, Spain; 5 Radiation Oncology Department, Institut Català d’Oncologia (ICO), L’Hospitalet de Llobregat, Spain; 6 Radiology Department, Hospital Universitari de Bellvitge (HUB), L’Hospitalet de Llobregat, Spain; 7 Pathology Department, Hospital Universitari de Bellvitge (HUB), L’Hospitalet de Llobregat, Spain; 8 Instituto de Investigación Biomédica de Bellvitge (IDIBELL), L’Hospitalet de Llobregat, Spain

**Keywords:** chordoma, immunotherapy, rare tumors, sarcoma, survival outcomes, treatment response, tyrosine kinase inhibitors

## Abstract

**Introduction:**

Chordoma is a rare malignant tumor originating in the notochord characterized by slow progression but frequent recurrences. Systemic treatment for this condition is not well defined. This study aimed to describe real-world clinical practice patterns of systemic therapy and its outcomes in patients with advanced chordoma treated at a sarcoma referral center member of the European Reference Network on Rare Adult Solid Cancers (EURACAN).

**Methods:**

Consecutive adult patients with histologically confirmed chordoma, diagnosed between 2005 and 2024, who received tyrosine kinase inhibitors (TKI) or immune checkpoint inhibitors (ICI), were retrospectively reviewed. Demographic, clinicopathological, and treatment data were collected from institutional databases. Responses were radiologically assessed according to RECIST criteria by sarcoma radiologists as part of routine clinical care. Data were collected up to December 31, 2024.

**Results:**

A total of 13 patients (median age 62 years) were identified. All had undergone surgery, and more than half received adjuvant radiation therapy. Most patients (n=10, 76.9%) received systemic therapy with imatinib as first-line treatment, while a minority (n=2, 15.4%) received ICIs as first-line therapy. Several patients received multiple lines of treatment, including sequential exposure to TKI and ICI. Objective responses were observed in 2 of 5 patients in the TKI-only subgroup (40.0%) and 4 of 8 patients in the ICI-exposed subgroup (50.0%), all of which were partial responses, with prolonged disease stabilization being the a common outcome. The median progression-free survival (PFS) for the entire cohort was 12.3 months, and the median overall survival (OS) was 149.8 months. The median PFS and median OS in the TKI-only subgroup were 7.4 and 113.5 months, respectively, whereas they were 12.7 and 151.6 months in the ICI-exposed subgroup, respectively. Subgroup results are descriptive, exploratory, and hypothesis-generating due to the small sample size.

**Conclusion:**

Our results indicate that systemic therapy can provide durable disease control in selected patients with chordoma. TKI are commonly used and may provide good responses while ICIs show potential activity in selected patients but await confirmation in robust clinical trials. These real-world data reinforce the need for prospective, multicenter studies to optimize treatment sequencing and patient selection in this rare malignancy.

## 1 Introduction

Chordoma is a rare malignant tumor that arises from the embryonic remnants of the notochord. It almost always arises along the axial skeleton, the sacrum, skull base, and mobile spine being the most common locations (1, 2). A review of population-based studies showed an estimated incidence of only 0.08 to 0.5 cases per 100,000 people per year, demonstrating its rarity and exceptionality even within the spectrum of bone malignancies (3, 4). However, despite representing only 1% to 4% of all primary bone tumors and approximately 5% of malignant bone tumors, chordoma has a significant clinical impact and morbidity due to its often-complex anatomical location and its propensity for local invasion and recurrence (5, 6).

Although chordomas tend to grow more slowly than other tumors, their behavior usually is insidiously aggressive. Despite radical surgery and adjuvant radiotherapy (RT), local recurrence remains common, and long-term disease control is difficult to achieve (2, 7). Advanced local treatment modalities such as proton beam or carbon ion therapy can be considered in order to optimize local control and minimize collateral damage to adjacent critical structures (8, 9). However, complete resections are often not feasible, and even high-dose RT cannot always prevent relapse or may be associated with local complications. Metastatic spread is less common than in other sarcomas, but once present, it is associated with limited therapeutic options and a poor prognosis (1, 7). Conventional systemic chemotherapy (ChT) has shown negligible benefit, and to date, no systemic ChT has become an established standard of care (7, 10).

Thus, other therapeutic strategies have been explored. The most investigated therapies have been tyrosine kinase inhibitors (TKI). Among these, imatinib was assessed in a prospective phase II trial. A total of 50 Response Evaluation Criteria in Solid Tumors (RECIST)-evaluable patients with advanced chordoma treated with imatinib achieved an objective response rate (ORR) of only 2%, although approximately 70% experienced stable disease, with a clinical benefit rate (complete/partial response or stable disease ≥6 months) of 64%. The median progression-free survival (PFS) was approximately 9 months. Median overall survival (OS) has been reported as 34 months in prospective and retrospective series (10, 11). Another multitarget TKI with good results is sorafenib, evaluated in a phase II trial by the French Sarcoma Group, which demonstrated an ORR of 3.7%, a 9-month PFS rate of 73%, and a 12-month OS rate of 86.5%, showing clinically significant disease stabilization in a subgroup of patients (12). These results illustrate both the potential and limitations of TKI: whereas they can provide temporary disease control, they rarely induce durable responses, and their use remains off-label.

More recently, immune checkpoint inhibitors (ICI) have shown potential promising activity in chordoma, although their role in routine practice remains unclear. In a key report, Migliorini et al. described 3 patients with recurrent chordoma treated with ICI after failure of conventional therapies, achieving durable tumor stabilization or partial responses, and 1 patient remained progression-free for more than 12 months (13). A more recent single-patient case report detailed a patient with metastatic chordoma with a *PBRM1* A1209fs mutation who was treated with pembrolizumab and achieved a PFS of 9.3 months (14). In a review of treatment-refractory chordomas, Alexander et al. identified 8 case reports where programmed death-1/programmed death-ligand 1 (PD-1/PD-L1) inhibitors (sometimes combined with TKI or vaccine approaches) were used, with variable results: some patients had partial responses or prolonged disease stabilization, but not a consistent pattern of durable responses was found (15). In addition, a recent analysis of immune checkpoint expression and tumor immune microenvironment in chordomas revealed upregulation of PD-1, cytotoxic T-lymphocyte-associated protein 4 (CTLA-4), and other immune checkpoint genes, and selective enrichment of memory T cells and macrophages, suggesting a biological rationale for their therapeutic targeting (16).

Furthermore, a recent phase I multicenter clinical trial evaluated the anti–PD-L1 monoclonal antibody FAZ053, alone or combined with spartalizumab (anti–PD-1), in patients with advanced malignancies, including a chordoma cohort. In that expansion cohort, FAZ053 monotherapy achieved an ORR of 30% (1 complete and 2 partial responses) and disease control in 70% of patients, with a manageable toxicity profile. In addition, biomarker analyses showed increased CD8+ infiltration and immune gene upregulation after treatment (17), providing prospective evidence of meaningful clinical activity of PD-(L)1 blockade in chordoma. There is also growing interest in combination immunomodulatory strategies: one report described the use of AdAPT-001 (a viral vector that delivers a TGF-β trap) to sensitize a recurrent chordoma to checkpoint inhibition, with evidence of radiographic tumor shrinkage (18). Taken together, these early experiences support the hypothesis that immunotherapy can achieve tumor control in a subset of chordomas. Definitive proof of its benefit, biomarkers of response, and optimal combination strategies remain to be established.

Given the challenges and the lack of solid evidence for systemic treatments, the management of advanced and metastatic chordoma continues to rely on multidisciplinary expertise, individualized decision-making, and participation in clinical trials, the latter of which are scarce. Therefore, we conducted a retrospective, real-world study in order to achieve a deeper understanding of treatment outcomes in clinical practice, including response patterns, recurrence dynamics, and survival.

## 2 Material and methods

### 2.1 Study design and patients

We conducted a retrospective single-center study at the Institut Català d’Oncologia (ICO)/Hospital Universitari de Bellvitge (HUB), Barcelona, Spain, a European Reference Network on Rare Adult Solid Cancers (EURACAN) member center. Consecutive adult patients (≥18 years) with histologically confirmed chordoma diagnosed between January 2005 and December 2024, metastatic at diagnosis or not, were identified from institutional databases. Follow-up data were collected until December 31, 2024.

Patients were eligible if they had received systemic therapy with TKI and/or ICI (anti–PD-1 or anti–PD-L1 agents). Among 15 initially identified patients, two were excluded due to incomplete clinical information related to the transition from paper to electronic medical records between 2005 and 2010. The final study cohort therefore consisted of 13 patients.

**Figure 1:**
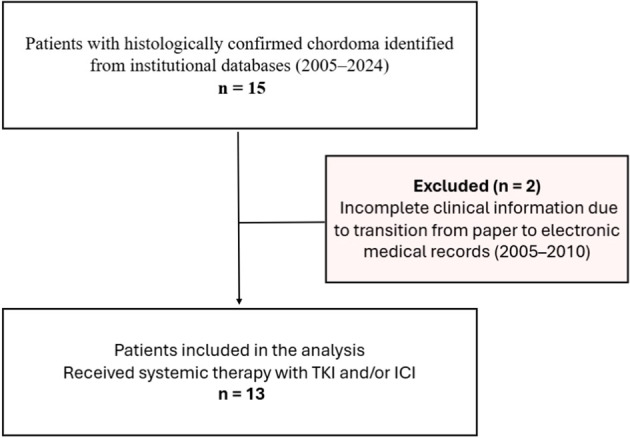
Patient selection flowchart. Flow diagram illustrating the identification, exclusion, and inclusion of patients in the study cohort. Fifteen patients with histologically confirmed chordoma were identified from institutional databases between 2005 and 2024. Two patients were excluded due to incomplete clinical information related to the transition from paper to electronic medical records. The final study cohort consisted of 13 patients who received systemic therapy with TKI and/or ICI.

Demographic, clinical, pathological, treatment, and follow-up data were extracted from electronic medical records. A flow diagram summarizing patient identification and selection is provided in **Figure 1**.

### 2.2 Local treatment

All patients underwent surgery at diagnosis, with or without subsequent RT depending on residual disease status and multidisciplinary team recommendation. Radical-intent resections, presence of residual disease, and use of adjuvant RT were recorded. Local relapses were defined as radiological or pathological recurrence at the primary site or regional compartments following initial therapy.

### 2.3 Systemic therapy

Systemic treatments included TKI and ICI, administered either within clinical trials or as compassionate use. For patients receiving multiple lines, treatment sequences and combinations were documented.

### 2.4 Outcomes

Outcomes of interest include ORR, disease control rate (DCR), duration of treatment (DOT), PFS and OS. Tumor response was assessed radiologically according to RECIST criteria, and radiological assessments were performed prospectively as part of routine clinical care by sarcoma-specialized radiologists. For the purposes of this retrospective study, RECIST-based response data were extracted from clinical radiology reports. No additional image re-evaluation, centralized review, or blinded assessment was performed.

ORR was defined as the proportion of patients achieving a complete or partial response (best overall response during systemic therapy). DCR was defined as the proportion of patients achieving complete response, partial response, or stable disease. DOT was defined as the time from treatment initiation to definitive discontinuation. Temporary treatment interruptions were included in DOT, whereas treatment re-challenge after documented progression was considered a new treatment course.

Relapse after radical-intent local therapy was recorded as the first radiologically or pathologically confirmed recurrence at local or distant sites. PFS was defined as the time from initiation of the systemic treatment defining the patient subgroup (TKI for the TKI-only group, and ICI for the ICI-exposed group) to documented disease progression or death from any cause, regardless of the treatment line in which it was administered. OS was calculated from the date of diagnosis to death from any cause or last follow-up.

For subgroup analyses, patients were classified according to exposure to ICI at any time during their disease course. Patients who never received ICI were categorized as the TKI-only group, whereas those who received ICI at any time were categorized as the ICI-exposed group, regardless of treatment sequence or combination. All survival and response analyses were performed at the patient level.

Patients without documented disease progression or death at the time of analysis were censored at the date of last clinical or radiological follow-up. Patients alive at last follow-up were censored for overall survival. No patients were lost to follow-up.

### 2.5 Statistical analysis

Descriptive statistics were used to summarize baseline characteristics. Continuous variables were reported as medians and ranges, and categorical variables as absolute numbers and percentages. Survival probabilities were estimated using the Kaplan–Meier method with 95% confidence intervals (CI). Given the small sample size, all patient-level subgroups (TKI-only vs ICI-exposed) comparisons were purely descriptive and exploratory in nature and should be interpreted as hypothesis-generating. Analyses were performed using R software, version 4.3.1 (R Foundation for Statistical Computing, Vienna, Austria).

### 2.6 Ethics

This study was conducted in accordance with the Declaration of Helsinki and applicable local regulations. Given its retrospective, non-interventional design, the use of fully anonymized clinical data, and the absence of biological sample collection, formal approval by an institutional review board or ethics committee was not required according to local regulations. In accordance with institutional policy for retrospective anonymized studies, formal IRB review was not required. Informed consent was waived.

## 3 Results

### 3.1 Patient characteristics and local treatment

A total of 13 patients were included. The median age at diagnosis was 62 years (range, 39–83), and most were female (n=8, 61.5%). Conventional histology predominated (n=11, 84.6%), and the sacrococcygeal region was the most frequent primary tumor site (n=6, 46.1%). All patients underwent initial local surgery, more than half received adjuvant RT (n=7, 53.8%), and nearly all experienced multiple relapses during their disease course (n=11, 84.6%). Detailed baseline and treatment characteristics are summarized in **Table 1**. Baseline characteristics according to patient-level treatment subgroup are summarized in **Supplementary Table T1**. Given the small sample size and treatment sequencing, subgroup comparisons are descriptive only.

**Table 1:** Baseline patient and treatment characteristics (n=13).

Characteristic	Value
Age, years — median (range)	62 (39–83)
Female sex — n (%)	8 (61.5)
Histologic subtype	
• Conventional — n (%)	11 (84.6)
• Other — n (%)	2 (15.4)
Primary site	
• Sacrococcygeal — n (%)	6 (46.1)
• Clivus — n (%)	3 (23.1)
• Vertebral — n (%)	2 (15.4)
• Extraaxial — n (%)	2 (15.4)
Residual disease post-surgery — n (%)	3 (23.1)
Adjuvant radiotherapy — n (%)	7 (53.8)
≥3 relapses — n (%)	11 (84.6)

Percentages are calculated using the total number of patients (n=13) as the denominator.

### 3.2 Systemic therapy

Systemic treatment was administered across heterogeneous clinical scenarios and treatment lines. Of the 13 patients included in the study, eight (61.5%) were exposed to ICI at some point during their disease course, whereas five patients (38.5%) received TKI only.

Ten patients (76.9%) initiated systemic therapy with a TKI—imatinib in all cases—while a minority received an ICI (n = 2, 15.4%). One patient (7.7%) received anthracycline-based ChT, as he was initially misdiagnosed with high-grade pleomorphic sarcoma and was later reclassified as chordoma after pathology review. ICI were most commonly introduced after prior TKI exposure, although in selected cases they were used upfront. Two patients (15.4%) underwent multiple ICI courses, including re-challenge. All ICI administered outside of clinical trials corresponded to atezolizumab.

Detailed systemic treatment distributions are provided in **Table 2** and treatment sequencing and line-level systemic therapy exposure for individual patients are summarized in **Supplementary Table T2**.

**Table 2:** Systemic therapy lines, sequences, and access in the study cohort (n=13).

Domain	Category	n (%)
First-line systemic regimen	Imatinib (TKI)	10 (76.9)
	ICI (clinical trial)	2 (15.4)
	Doxorubicin + olaratumab (anthracycline-based ChT)	1 (7.7)
Number of systemic therapy lines per patient	1 line	6 (46.1)
	2 lines	3 (23.1)
	3 lines	4 (30.7)
Exposure categories	TKI only	5 (38.5)
	ICI only	2 (15.4)
	Both TKI and ICI (sequential)	6 (46.1)
Sequence patterns	TKI → ICI (± other)	6 (46.1)
	ICI only	2 (15.4)
	TKI only	5 (38.5)
ICI access route	Compassionate use only	5 (62.5)
	Clinical trial only	1 (12.5)
	Both (compassionate + trial)	2 (25.0)
Additional TKI post-ICI	Sorafenib	1 (7.7)

All ICI administered outside of clinical trials corresponded to atezolizumab. Percentages are calculated using the total number of patients (n=13) as the denominator, unless otherwise specified. For ICI access route, percentages are calculated among ICI-exposed patients (n=8).

TKI, tyrosine kinase inhibitor; ICI, immune checkpoint inhibitor; ChT, chemotherapy.

### 3.3 Outcomes

In the TKI-only subgroup (n=5), best overall response during systemic therapy was partial response (PR) in 2 patients, stable disease (SD) in 2, and progressive disease (PD) in 1, yielding an ORR of 40.0%. In the ICI-exposed subgroup (n=8), best overall response was PR in 4 patients and SD in 4, resulting in an ORR of 50.0%. No complete responses were observed in either subgroup. Disease control rates are summarized in **Table 3**. Median DOT was 12.4 months (95% CI, 4.3–20.6) overall; 7.6 months (95% CI, 2.5–12.7) in the TKI-only subgroup; and 12.5 months (95% CI, 4.0–20.2) in the ICI-exposed subgroup.

**Table 3:** Objective responses to systemic therapy according to patient-level treatment subgroup (n=13).

Treatment group	Patients treated, n	Partial responses, n (%)	Complete responses, n (%)	ORR, %	DCR, %
TKI-only subgroup	5	2 (40.0)	0 (0)	40.0	80.0
ICI-exposed subgroup	8	4 (50.0)	0 (0)	50.0	100.0

Subgroups are defined according to exposure to ICI at any time during the disease course. Percentages are calculated using the number of patients in each subgroup as the denominator.

TKI, tyrosine kinase inhibitor; ICI, immune checkpoint inhibitor; ORR, objective response rate; DCR, disease control rate.

Median OS and PFS are reported for descriptive purposes only. OS was calculated from the date of diagnosis and therefore does not share the same time zero as PFS, which was defined from the initiation of the subgroup-defining systemic therapy. Accordingly, OS estimates should not be interpreted as comparative measures of treatment effect between subgroups.

**Figure 2:**
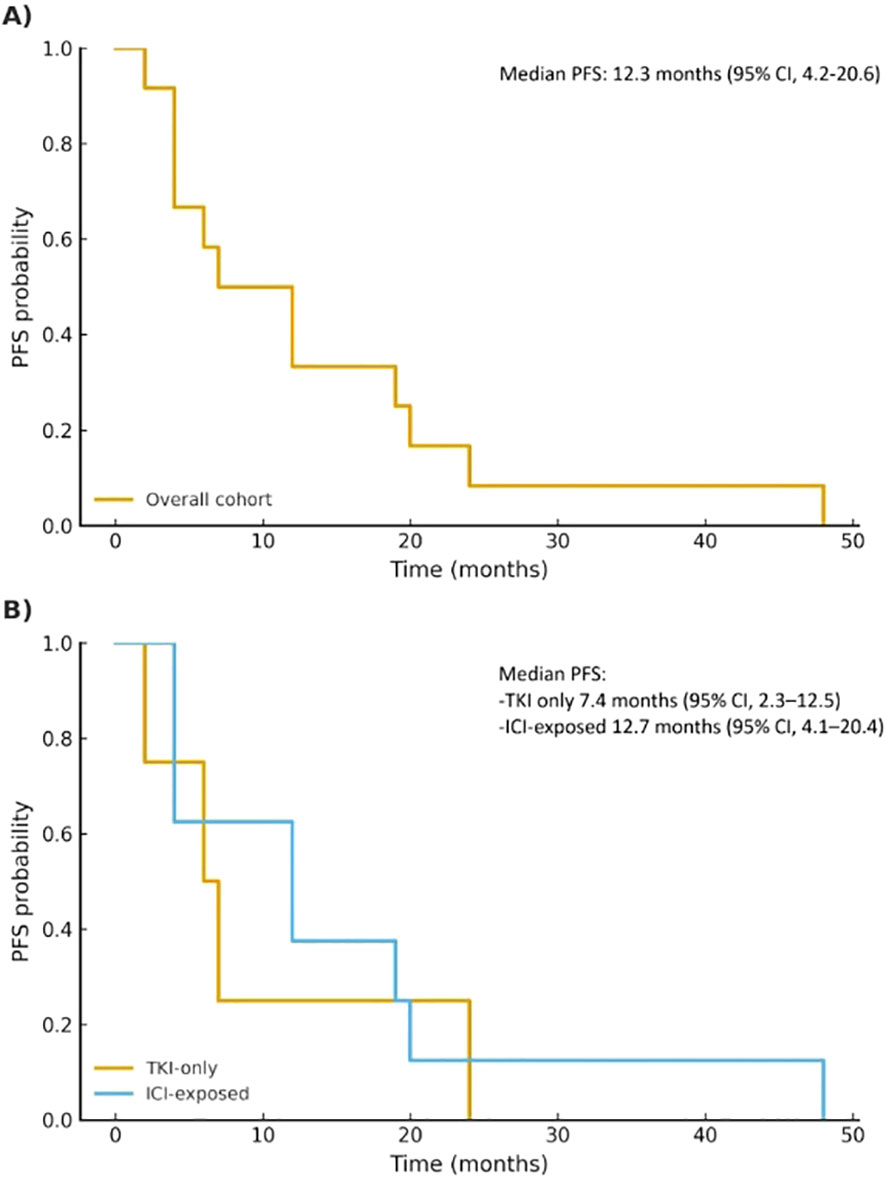
Kaplan–Meier curves showing PFS for the entire cohort **(A)** and stratified by patient-level treatment subgroup **(B)**. Median estimates with 95% confidence intervals are shown alongside each panel. Due to the small sample size, number-at-risk tables and censoring marks are not shown.

Median PFS for the entire cohort was 12.3 months (95% CI, 4.2–20.6). When stratified by patient-level treatment subgroup, median PFS was 7.4 months (95% CI, 2.3–12.5) in the TKI-only subgroup (n=5), and 12.7 months (95% CI, 4.1–20.4) in the ICI-exposed subgroup (n=8; patients who received at least one line of ICI, with or without prior TKI) (**Figure 2**).

**Figure 3:**
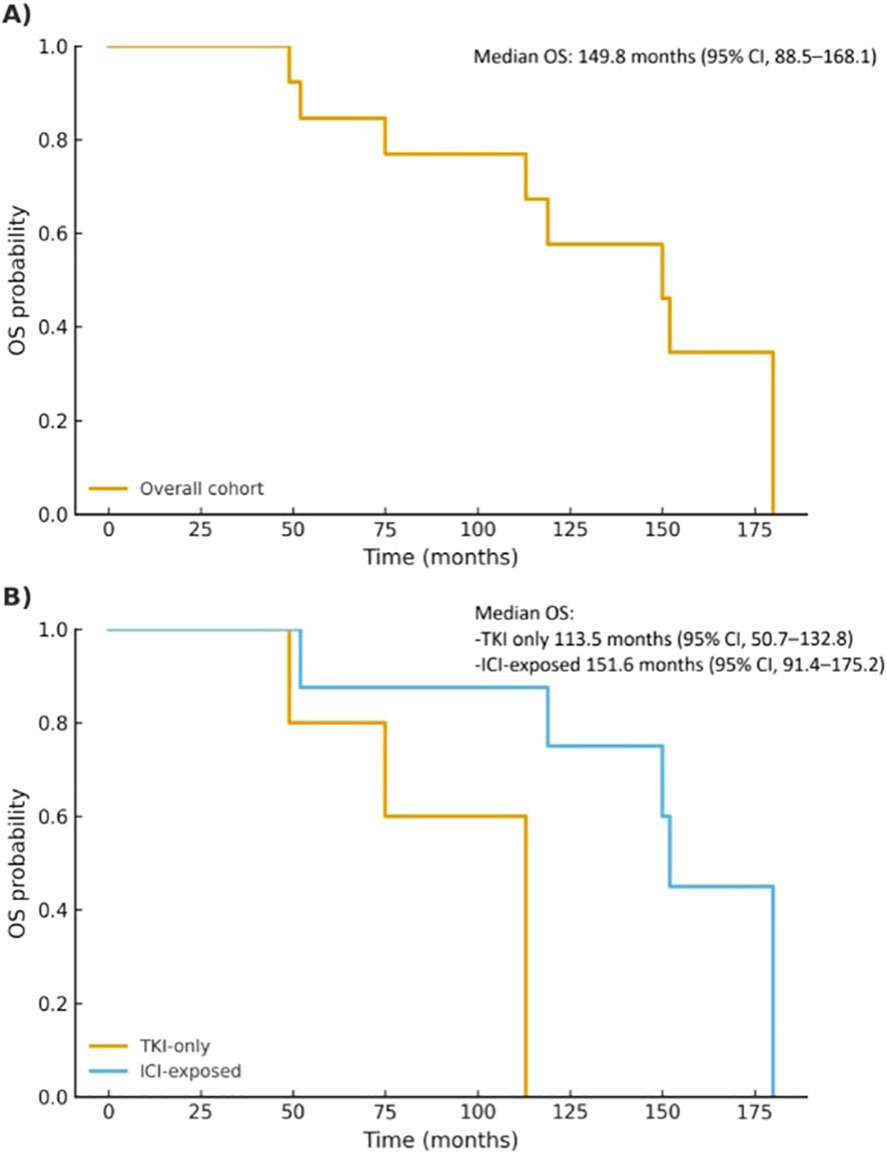
Kaplan–Meier curves showing OS for the entire cohort **(A)** and stratified by patient-level treatment subgroup **(B)**. Median estimates with 95% confidence intervals are shown alongside each panel. Due to the small sample size, number-at-risk tables and censoring marks are not shown.

Median OS for the whole series was 149.8 months (95% CI, 88.5–168.1). Median OS reached 113.5 months (95% CI, 50.7–132.8) in the TKI-only subgroup (n=5) and 151.6 months (95% CI, 91.4–175.2) in the ICI-exposed subgroup (n=8) (**Figure 3**).

## 4 Discussion

Our single-center series reflects real-world, non-standardized treatment sequencing in a rare disease, shaped by availability of clinical trials, compassionate use access, and evolving evidence. The clinical characteristics of our cohort are consistent with those previously reported in other prospective and retrospective studies at referral centers. Patients were generally around 60 years old, conventional histology predominated, and the sacrococcygeal site was the most common primary site. Although all patients underwent surgery for the primary lesion and more than half received adjuvant RT, relapse was widespread, with most patients experiencing multiple local or systemic recurrences requiring therefore a systemic approach. This pattern is consistent with the indolent but recurrent behavior of chordoma and explains why many patients eventually exhaust local treatment options and require systemic therapy.

From a systemic treatment perspective, our cohort shows the therapeutic diversity of chordoma treatment, mainly due to the lack of standard therapeutic guidelines with robust evidence. Most patients received imatinib as first-line treatment, following the limited evidence available, while a few initiated ICI therapy. One patient received initial treatment with anthracycline-based ChT after an initial misdiagnosis of soft tissue sarcoma, illustrating how histological ambiguity can influence management in rare malignancies. Nearly half of the patients received more than 1 line of systemic therapy, including rechallenges with ICI and sequential transitions between TKI and ICI. Furthermore, 1 patient received sorafenib after immunotherapy. Treatment allocation was not randomized, and patients exposed to ICI likely represent a selected subgroup treated at later disease stages, which may introduce selection bias in subgroup comparisons. This heterogeneity is an example of the daily clinical reality in infrequent tumors without established standard treatment algorithms. Detailed and standardized toxicity data could not be systematically analyzed due to the long study period and the transition from paper-based to electronic medical records, which limited the reliability and completeness of adverse event reporting.

Despite this diversity, the observed therapeutic outcomes were in line with previously reported data. Objective responses occurred with both drug classes. The median overall PFS in our series was 12.3 months, slightly longer than the approximately 9 months reported in the pivotal phase II study with imatinib, while the median OS was long, exceeding 10 years. Moreover, our data adds to the heterogeneous but growing evidence of chordomas treated with ICI from case reports and clinical trials. Partial responses with anti-PD(L)1 drugs, as seen here, were also documented by Migliorini et al. (13) and in the recent phase I FAZ053 trial (17). The median PFS observed in our ICI-exposed patients is consistent with previously published data reported with pembrolizumab monotherapy (13, 14). These findings should be interpreted with caution, as numerically longer survival observed in the ICI-exposed subgroup may reflect treatment activity but is also likely influenced by selection bias, given that patients receiving immunotherapy within clinical trials are typically highly selected and may have more favorable prognostic features. Overall, these results suggest that systemic therapy may influence the disease course in a subset of patients.

Several limitations of this study should be acknowledged. Heterogeneity in systemic treatment exposure, including differences in agents used, treatment sequencing, and lines of therapy, introduces potential confounding. Given the retrospective design and small sample size, subgroup analyses are descriptive and hypothesis-generating. OS should be interpreted with caution, as it was measured from the date of diagnosis, whereas subgroup PFS was defined from the initiation of the subgroup-defining systemic therapy, resulting in different time-zero definitions. Together with the small sample size, the limited number of death events, and the indolent natural history of chordoma, this precludes meaningful comparative interpretation of OS between subgroups. In addition, competing risks such as non–cancer-related death may influence OS outcomes. Accordingly, OS estimates are presented to provide longitudinal clinical context and should be interpreted as descriptive and hypothesis-generating rather than reflective of treatment-related effects. The definition of the ICI-exposed subgroup is time-dependent and may be affected by immortal time bias. In addition, molecular profiling data were not systematically available, and radiological response assessments were retrospectively extracted from routine clinical reports without centralized or blinded review. These limitations underscore that the findings of this study are descriptive rather than confirmatory.

This study adds to the existing literature by providing single-center real-world data on systemic therapy outcomes in advanced chordoma across a long historical period, including both TKI and ICI exposure within the same institutional cohort. Unlike clinical trials or pooled retrospective analyses, our series reflects routine treatment sequencing, patient selection, and follow-up in daily clinical practice. These data are intended to complement, rather than directly compare with, previously published studies and should be interpreted as hypothesis-generating for future research.

## 5 Conclusion

Our study describes the clinical reality of chordoma in a referral center for sarcomas: a malignancy with a slow but persistent course, with frequent recurrences despite local treatment with surgery and RT, and non-standardized systemic therapy. TKI remain a rational first-line option for indolent disease, often achieving prolonged disease stabilization, while ICI may represent a promising therapeutic strategy capable of inducing meaningful responses in selected patients. Given the absence of standardized treatment sequencing and predictive biomarkers, prospective multicenter collaboration is needed to harmonize therapeutic strategies, refine patient selection, and define the role of immunotherapy in this rare and challenging disease.

## Data Availability

The raw data supporting the conclusions of this article will be made available by the authors, without undue reservation.

## Supplementary material

Supplementary Table 1 Baseline characteristics according to patient-level treatment subgroup. Comparisons are descriptive only due to the small sample size. No formal statistical testing was performed. Percentages are calculated using the number of patients in each subgroup as the denominator.

Supplementary Table 2 Patient-level systemic treatment sequencing and line-level outcomes. Systemic therapy lines refer to consecutive lines of systemic treatment administered after local therapy. All ICI administered outside of clinical trials corresponded to atezolizumab. Best response was assessed according to RECIST criteria and corresponds to the best overall response achieved during each systemic treatment line. The dashed line separates patients treated with TKI only from those exposed to ICI at any time during the disease course.
