# Supplementary material for: Immunotherapy and tyrosine kinase inhibitors in chordoma: a real-world data study from a European Reference Network on Rare Adult Solid Cancers member center
Source: Front Oncol. 2026 Feb 3;16:1755538. doi: 10.3389/fonc.2026.1755538 (PMC12909182; doi:10.3389/fonc.2026.1755538)
Supplement: Supplementary Table 1 — Baseline characteristics according to patient-level treatment subgroup. Comparisons are descriptive only due to the small sample size. No formal statistical testing was performed. Percentages are calculated using the number of patients in each subgroup as the denominator. [file Table1.docx]

**Supplementary Table T1**. **Baseline characteristics according to patient-level treatment subgroup.** Comparisons are descriptive only due to the small sample size. No formal statistical testing was performed. Percentages are calculated using the number of patients in each subgroup as the denominator.

| **Characteristic** | **TKI-only (n=5)** | **ICI-exposed (n=8)** |
| --- | --- | --- |
| Age at diagnosis — median (range) | 51 (37–78) | 59 (44–66) |
| Sex | | |
| - Female — n (%) | 3 (60.0) | 5 (62.5) |
| - Male — n (%) | 2 (40.0) | 3 (37.5) |
| Primary tumour location | | |
| - Sacrococcygeal — n (%) | 2 (40.0) | 4 (50.0) |
| - Clivus — n (%) | 3 (60.0) | 0 (0) |
| - Vertebral — n (%) | 0 (0) | 2 (25.0) |
| - Extraaxial — n (%) | 0 (0) | 2 (25.0) |
| Prior primary tumor surgery — n (%) | 5 (100.0) | 8 (100.0) |
| Residual disease post-surgery — n (%) | 1 (20.0) | 2 (25.0) |
| Adjuvant radiotherapy — n (%) | 3 (60.0) | 4 (50.0) |
| Line of systemic therapy at subgroup-defining treatment — n (%) | | |
| First line — n (%) | 5 (100.0) | 2 (25.0) |
| ≥ Second line — n (%) | 0 (0) | 6 (75.0) |

**Supplementary Table T2**. **Patient-level systemic treatment sequencing and line-level outcomes.** Systemic therapy lines refer to consecutive lines of systemic treatment administered after local therapy. All ICI administered outside of clinical trials corresponded to atezolizumab. Best response was assessed according to RECIST criteria and corresponds to the best overall response achieved during each systemic treatment line. The dashed line separates patients treated with TKI only from those exposed to ICI at any time during the disease course.

Abbreviations: TKI, tyrosine kinase inhibitor; ICI, immune checkpoint inhibitor; RT, radiotherapy; PR, partial response; SD, stable disease; PD, progressive disease; ChT, chemotherapy.

| **Patient** | **Primary site** | **Metastasis at diagnosis** | **Primary tumor surgery** | **Adjuvant RT** | **ICI exposure** | **1^st^ systemic therapy; best response** | **2^nd^ systemic therapy; best response** | **3^rd^ systemic therapy; best response** | **4^th^ systemic therapy; best response** |
| --- | --- | --- | --- | --- | --- | --- | --- | --- | --- |
| **1** | Clivus | No | Yes | Yes | No | Imatinib; PD |  |  |  |
| **2** | Clivus | No | Yes | No | No | Imatinib; SD |  |  |  |
| **3** | Sacrococcygeal | No | Yes | No | No | Imatinib; SD |  |  |  |
| **4** | Sacrococcygeal | No | Yes | Yes | No | Imatinib; **PR** |  |  |  |
| **5** | Clivus | No | Yes | Yes | No | Imatinib; **PR** |  |  |  |
| **6** | Sacrococcygeal | No | Yes | No | Yes | ChT; SD | Imatinib; PD | ICI; SD |  |
| **7** | Sacrococcygeal | No | Yes | No | Yes | ICI (clinical trial); SD | ICI; SD |  |  |
| **8** | Vertebral | No | Yes | Yes | Yes | Imatinib; SD | ICI; SD |  |  |
| **9** | Sacrococcygeal | No | Yes | Yes | Yes | ICI; **PR** |  |  |  |
| **10** | Vertebral | No | Yes | Yes | Yes | Imatinib; SD | ICI; SD |  |  |
| **11** | Sacrococcygeal | No | Yes | No | Yes | Imatinib; PR | ICI; **PR** | ICI (clinical trial); **PR** |  |
| **12** | Extraaxial | Yes, pleural | Yes | No | Yes | Imatinib; SD | ICI (clinical trial); **PR** | ICI (clinical trial); SD |  |
| **13** | Extraaxial | No | Yes | Yes | Yes | Imatinib; PD | ICI; **PR** | Sorafenib; PD |  |
